# Dose Enhancement for the Flattening-Filter-Free and Flattening-Filter Photon Beams in Nanoparticle-Enhanced Radiotherapy: A Monte Carlo Phantom Study

**DOI:** 10.3390/nano10040637

**Published:** 2020-03-29

**Authors:** Stefano Martelli, James C L Chow

**Affiliations:** 1 Department of Physics, Ryerson University, Toronto, ON M5B 2K3, Canada; smartelli@ryerson.ca; 2 Department of Radiation Oncology, University of Toronto and Radiation Medicine Program, Princess Margaret Cancer Centre, University Health Network, Toronto, ON M5G 1X6, Canada

**Keywords:** dose enhancement, nanoparticle-enhancement radiotherapy, flattening-filter-free, flattering-filter, Monte Carlo simulation, nanoparticles

## Abstract

Monte Carlo simulations were used to predict the dose enhancement ratio (DER) using the flattening-filter-free (FFF) and flattening-filter (FF) photon beams in prostate nanoparticle-enhanced radiotherapy, with multiple variables such as nanoparticle material, nanoparticle concentration, prostate size, pelvic size, and photon beam energy. A phantom mimicking the patient’s pelvis with various prostate and pelvic sizes was used. Macroscopic Monte Carlo simulation using the EGSnrc code was used to predict the dose at the prostate or target using the 6 MV FFF, 6 MV FF, 10 MV FFF, and 10 MV FF photon beams produced by a Varian TrueBeam linear accelerator (Varian Medical System, Palo Alto, CA, USA). Nanoparticle materials of gold, platinum, iodine, silver, and iron oxide with concentration varying in the range of 3–40 mg/ml were used in simulations. Moreover, the prostate and pelvic size were varied from 2.5 to 5.5 cm and 20 to 30 cm, respectively. The DER was defined as the ratio of the target dose with nanoparticle addition to the target dose without nanoparticle addition in the simulation. From the Monte Carlo results of DER, the best nanoparticle material with the highest DER was gold, based on all the nanoparticle concentrations and photon beams. Smaller prostate size, smaller pelvic size, and a higher nanoparticle concentration showed better DER results. When comparing energies, the 6 MV beams always had the greater enhancement ratio. In addition, the FFF photon beams always had a better DER when compared to the FF beams. It is concluded that gold nanoparticles were the most effective material in nanoparticle-enhanced radiotherapy. Moreover, lower photon beam energy (6 MV), FFF photon beam, higher nanoparticle concentration, smaller pelvic size, and smaller prostate size would all increase the DER in prostate nanoparticle-enhanced radiotherapy.

## 1. Introduction

There are three major methods of treating tumors, namely, radiotherapy, chemotherapy, and surgery. The goal of radiotherapy is to use the ionizing radiation to damage the deoxyribonucleic acid (DNA) of the cancerous cells so that they may undergo mitotic death [1,2]. Mitotic death is the most common form of cell death caused by radiation; it occurs when cells die while in the process of mitosis due to damaged chromosomes. Recently, many studies focused on the development of radiosensitizers such as heavy-atom nanoparticles in radiotherapy [3,4,5,6,7,8]. These nanomaterials aggregate in the cell because of the interactions with glutathione present in cytosol. Since the level of glutathione in cancer cells is much higher than that in normal cells, heavy-atom nanoparticles are found accumulated more in tumors compared to normal tissue [9,10]. Therefore, to enhance the radiation treatment while protecting the surrounding normal tissues, nanoparticles can be placed in the tumorous regions to maximize the dose deposition. The dose enhancement ratio (DER) is defined as the ratio of the target dose with the nanoparticle addition to the target dose without the nanoparticles addition [4,11]. Having a higher DER allows for a better local tumor control by increasing the dose to the cancerous target, while exposing the healthy surrounding tissues to a lower and more tolerable dose. The probability of absorption for the photoelectric effect is proportional to the atomic number and inversely proportional to the photon energy. Therefore, nanoparticles having a high atomic number and a low energy photon irradiation will increase the probability of absorption to the target with nanoparticle addition [12,13].

In radiotherapy, a flattening-filter (FF) is used to flatten the photon beam to provide a uniform profile for a homogeneous dose distribution at the tumor. With the recent application of intensity-modulated radiotherapy using a multileaf collimator, the delivered beam profile can be controlled by the beam intensity using a combination of irregular beam segments generated by the collimator [14]. This means that the FF is unnecessary and can be taken out from the head of the linear accelerator. Therefore, a new type of flattening-filter-free (FFF) photon beam is generated, leading to a huge increase (2 to 4-fold) of dose rate and decrease of beam-on time [15]. Although the FFF photon beam may increase the patient’s skin dose due to the presence of low-energy photon [16,17], more patients can be treated within the same period of time, and some delivery uncertainties such as intrafractional organ or tumor motion can be reduced. Therefore, using a FFF photon beam would result in an increase of patient throughput and a more accurate delivery. 

When nanoparticles are added to the tumor, dose enhancement occurs due to an increase of compositional atomic number [18]. As there is almost no low-energy photon attenuation due to the removal of the FF in a FFF photon beam, the low-energy photon in the FFF beam would enhance the photoelectric interaction, leading to a higher dose enhancement [3]. Moreover, it is found that dose enhancement would be affected by other factors such as the nanoparticle material and concentration [11,18]. The aim of this study was to compare the FFF and FF photon beams amongst multiple variables such as nanoparticle material, nanoparticle concentration, photon beam energy, prostate size, and pelvic size in prostate nanoparticle-enhanced radiotherapy using a phantom. The DER values were calculated by Monte Carlo simulation using a macroscopic approach [19,20]. In this approach, the nanoparticle-added medium is considered as a mixture of gold and water [21]. Therefore, the simulation does not determine the dependence of DER on the nanoparticle size. 

## 2. Materials and Methods 

The prostate or target dose in a pelvic phantom was determined by Monte Carlo simulation using the EGSnrc-based code [22]. Phase-space files containing information of particle type, orientation, and location of photon beams of the 6 MV FFF, 6 MV FF, 10 MV FFF, and 10 MV FF were generated based on a Varian TrueBeam linear accelerator (Varian Medical System, Palo Alto, CA, USA). The head of the accelerator was modeled using the Geant4 Monte Carlo code [23]. Since the position of the scoring planes, in which the beam phase-space data were scored, were only above the jaws of the accelerator head using the Geant4 code, the BEAMnrc code [24] was used to generate the final phase-space file at the secondary collimator based on the phase-space data from the Geant4. The field size of the photon beam is equal to 10 × 10 cm^2^, and each phase-space file contained 1 × 10^9^ particles. The Monte Carlo model was verified by comparing the Monte Carlo dosimetry with measurements using a commissioning water tank and ionization chamber. The results of verification can be found elsewhere [16,17].

The material data library of the nanoparticles was generated using the EGSnrc-based PEGS code [19]. In this study, nanoparticle materials, which have proven experimental evidence and high potential to be applicable in radiotherapy were selected and investigated. These materials included gold [25], platinum [26], iodine [27], silver [28], and iron oxide (Fe_2_O_3_) [29]. The nanoparticle materials added to the soft tissue (water) were created with concentrations equal to 3, 7, 18, 30, and 40 mg/ml. These concentrations were set based on the small-animal experiment in nanoparticle-enhanced radiotherapy [30]. A pelvic phantom with a prostate in the center was used in the simulation [5]. The phantom size was varied at 20, 25, and 30 cm, and the prostate size was varied at 2.5, 3.5, 4.5, and 5.5 cm. The range of prostate and phantom sizes were set up based on our external beam treatment planning experience in prostate radiotherapy [31,32].

To calculate the DER with the FFF and FF photon beams and other nanoparticle and phantom variables, the pelvic phantom was irradiated by the 6 MV FFF, 6 MV FF, 10 MV FFF, and 10 MV FF photon beams with the prostate material set to soft tissue (water) only and soft tissue plus nanoparticles with different concentrations. The beam geometry and other Monte Carlo parameters were set to be the same when the nanoparticles were added or not added to the phantom.

The target (prostate) doses in the above settings were predicted using Monte Carlo simulations, and the DER was calculated as [33]:

(1) $$Dose\;Enhancement\;Ratio\;\left(DER\right)=\frac{Target\;dose\;with\;nanoparticle\;addition}{Target\;dose\;without\;nanoparticle\;addition}.$$

When there is no nanoparticle added to the pelvic phantom, the DER is equal to one according to Equation (1). The DER reflects the fractional increase of dose at the target (prostate) when nanoparticles are added in radiotherapy. Since the nanoparticle addition increases the compositional atomic number of the target, the DER value should be greater than one in nanoparticle-enhanced radiotherapy [12,13].

## 3. Results and Discussion 

**Figure 1:**
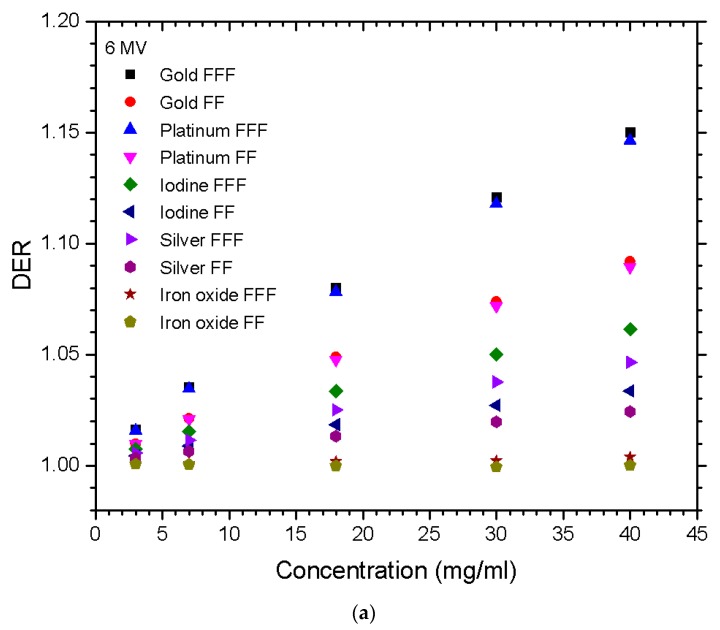

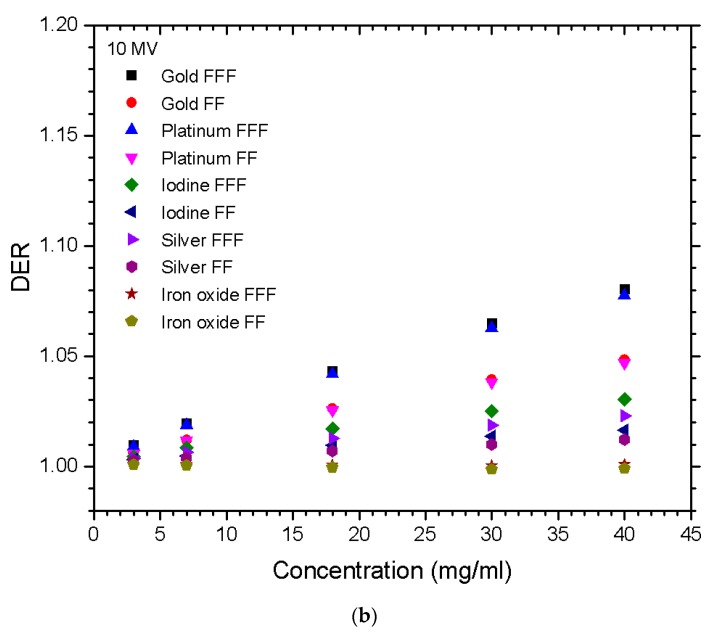
Relationships of the dose enhancement ratio (DER) and nanoparticle concentration vary with different materials using the (**a**) 6 MV and (**b**) 10 MV photon beams. Gold, platinum, iodine, silver, and iron oxide nanoparticles with concentrations equal to 3, 7, 18, 30, and 40 mg/ml were used. The DER was calculated as the ratio of the target dose with nanoparticle addition to the target dose without nanoparticle addition under the same simulation configuration.

**Figure 2:**
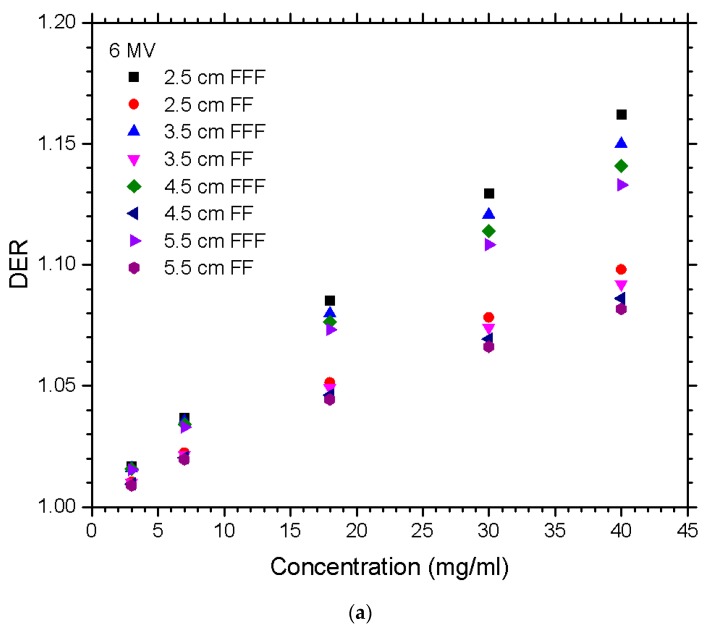

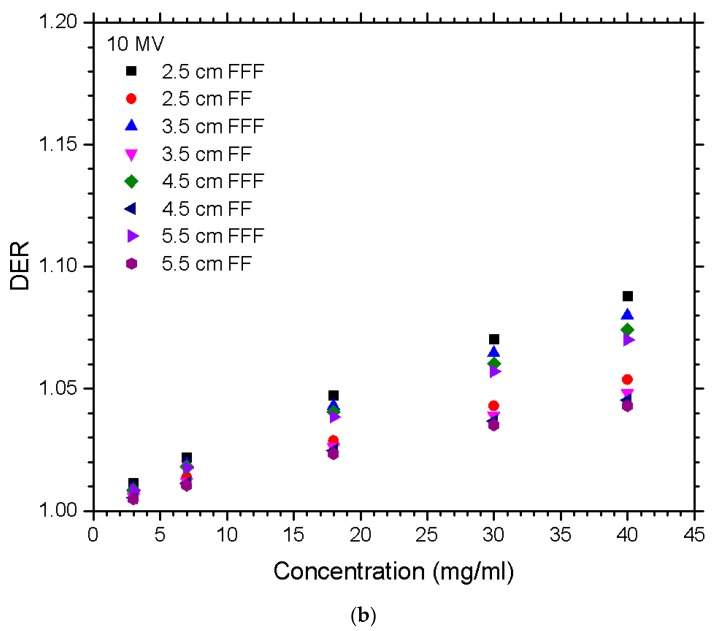
Relationships of the DER and nanoparticle concentration varying with different prostate sizes in the phantom using the (**a**) 6 MV and (**b**) 10 MV photon beams. Gold nanoparticles with concentrations equal to 3, 7, 18, 30, and 40 mg/ml were used. The DER was calculated as the ratio of the target dose with nanoparticle addition to the target dose without nanoparticle addition under the same simulation configuration.

**Figure 3:**
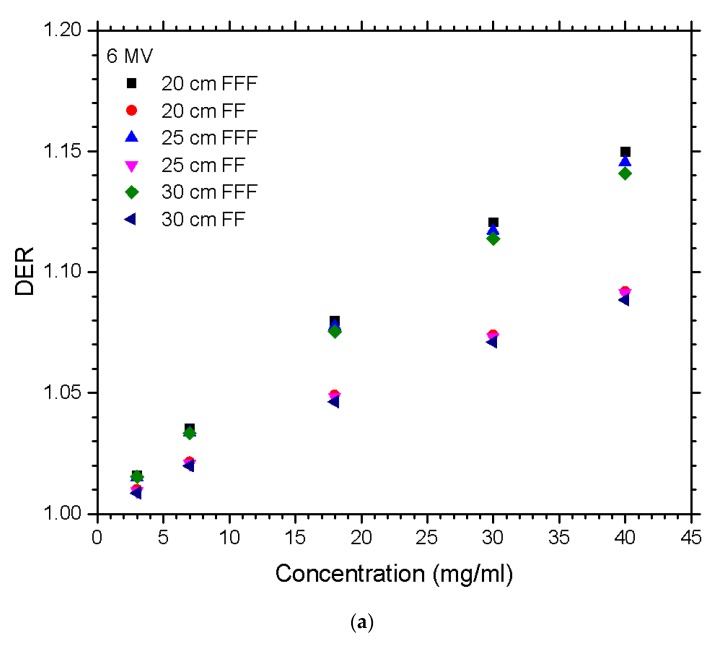

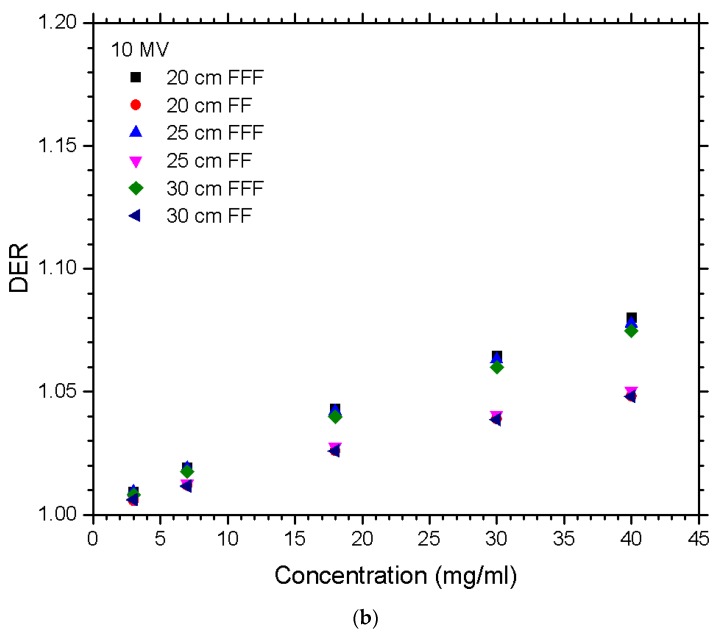
Relationships of the DER and nanoparticle concentration varying with different pelvic sizes using the (**a**) 6 MV and (**b**) 10 MV photon beams. Gold nanoparticles with concentrations equal to 3, 7, 18, 30, and 40 mg/ml were used. The DER was calculated as the ratio of the target dose with nanoparticle addition to the target dose without nanoparticle addition under the same simulation configuration.

**Figure 4:**
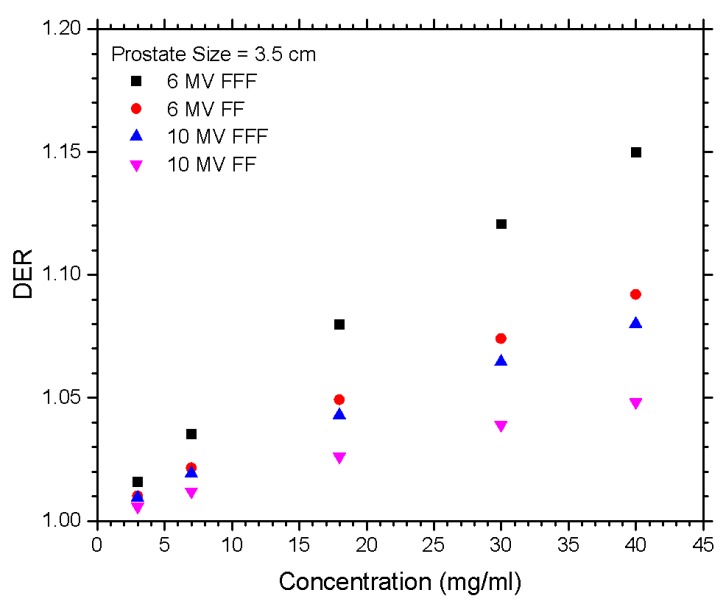
Relationships of the DER and gold nanoparticle concentration for the 6 MV FFF, 6 MV FF, 10 MV FFF, and 10 MV FF photon beams. The pelvic and prostate size were equal to 25 cm and 3.5 cm, respectively. The DER was calculated as the ratio of the target dose with nanoparticle addition to the target dose without nanoparticle addition under the same simulation configuration.

The relationships between the DER and nanoparticle concentration for different materials of gold, platinum, iodine, silver, and iron oxide, irradiated by the FFF and FF photon beams are shown in Figure 1a,b for beam energy equal to 6 and 10 MV. Figure 2a,b show the dependences of the DER on the gold nanoparticle concentration for various prostate sizes ranging from 2.5 to 5.5 cm using the 6 and 10 MV photon beams. The phantoms used in Figure 2 were irradiated by the FFF and FF photon beams for comparison. Dependences of the DER on the gold nanoparticle concentration for various phantom (pelvic) sizes ranging from 20 to 30 cm, using the 6 and 10 MV photon beams, are shown in Figure 3a,b, respectively. In addition, the relationships between the DER and the gold nanoparticle concentration for the 6 MV FFF, 6 MV FF, 10 MV FFF, and 10 MV FF photon beams are shown in Figure 4 in which the prostate and pelvic size were equal to 3.5 cm and 25 cm, respectively. 

### 3.1. Nanoparticle Material and Concentration

The results for the 6 MV photon beams on various nanoparticle concentrations and materials have a general trend, as shown in Figure 1a. It is noted that with all the nanoparticles except with iron oxide, an increase of concentration resulted in an increase of dose enhancement. This probably occurs because the more material present in the prostate with a higher atomic number, the more probable the photoelectric effect will happen for the low-energy photons in the beam [3]. It is worth noting the order from the lowest to highest DER of nanoparticles was as follows: iron oxide, silver, iodine, platinum, and gold. This also happened to be the same order from the lowest atomic number to the highest. The DER values from gold and platinum were very similar out of the other nanoparticles tested; this result is reasonable considering they only differ in atomic number by 1 and fall in the same period in the periodic table. Both iodine and silver being from the same period fell within close proximity in the DER. At 40 mg/ml on FFF, they only differed by 0.015, while comparing silver to gold, the difference was 0.104. Iron oxide with both the FFF and FF beams barely had any effect on the DER with increasing nanoparticle concentration. The results for the 10 MV photon beam on various nanoparticle concentrations and materials had very similar trends from the 6 MV beams, as shown in Figure 1b. One significant difference in the simulation using the 6 MV beam was the magnitude of the DER. When comparing Figure 1a,b, the 10 MV beam almost cut the DER value in half, most noticeably with gold and platinum. Iodine and silver also showed a lower DER when compared to the 6 MV results; however, there was not as much as gold and platinum. As for iron oxide, there was barely any growth as the nanoparticle concentration increased for either 6 MV or 10 MV beam energy. 

### 3.2. Prostate and Pelvic Size

For the 6 MV photon beam on different prostate sizes as shown in Figure 2a, it is seen that at smaller sizes, there was a greater DER as the nanoparticle concentration increased. All prostate sizes with FFF beams showed a greater DER when compared to the FF. At lower concentration, the difference was small; however, by the 40 mg/ml concentration, there was a significant increase in dose enhancement. This is because the FFF beam does not filter out the lower energy photons from getting to the nanoparticles and undergoing the photoelectric effect [34]. At the 3 and 7 mg/ml concentrations, the spread of the DER was almost negligible for the prostate size; however, at 18, 30, and 40 mg/ml, there were some noticeable differences. This happened for both the FFF and FF beams except that the FF had almost half of the DER as those with FFF. For the 10 MV photon beam on the different prostate sizes displayed in Figure 2b, it is found that at smaller sizes, there was a greater DER as the nanoparticle concentration increased. The results followed the same trend of the 6 MV beams; however, the dose enhancement for the 10 MV was only about half. In Figure 2, the maximum DER in the 6 MV FFF beam was 1.16 for the 2.5 cm prostate size, while the maximum DER in the 10 MV FFF beam was 1.08 with the same size of prostate.

In Figure 3a,b, it is seen that with an increasing gold nanoparticle concentration, the DER also increased. The DER values for the FFF photon beams were higher compared to those for the FF beams. With the FF beams presented, the results had an increasing DER as the gold nanoparticle concentration increased; however, the DER from the pelvic size was not very much spread. The FFF beam results showed that the smaller the pelvic size, the greater the DER value that was observed. As the gold nanoparticle concentration increased, the spread between dose enhancements was more noticeable. The overall significance was not massively different; however, there was one nonetheless. The simulation performed for the pelvic size on the 10 MV beams (Figure 3b) again showed a similar trend as that of the 6 MV in Figure 3a. In Figure 3, it can be seen that there was a higher DER value for the FFF beams compared to those of the FF. Moreover, the 10 MV beam DER values seemed to be about half the magnitude compared to the 6 MV: the maximum point on the 6 MV beam was 1.15, while for the 10 MV, it was 1.08.

### 3.3. Photon Beam Energy and FFF vs. FF 

Comparing the DER between the 6 and 10 MV beams showed a common trend as seen in Figure 4. Both of the 6 MV FFF and FF results showed a greater DER to any of the 10 MV values beyond the gold nanoparticle concentration of 18 mg/ml point. The 6 MV FF DER value was relatively close to the 10 MV FFF DER. The maximum DER at 40 mg/ml was 1.15 at 6 MV FFF, while for 10 MV FFF, it was 1.08. The nanoparticle material, prostate size, and pelvic size were all investigated for both the 6 and 10 MV, as seen in Figure 1–3. With all of them, there was a drop in the DER at the 10 MV beam when compared to the 6 MV beam, showing that lower energies were more suitable for maximizing the DER. This is because the 6 MV beam contained more low-energy photons considering its energy spectrum compared to the 10 MV beam [17,34].

## 4. Conclusions

In conclusion, this study throws light on how to maximize the DER in nanoparticle-enhanced radiotherapy. Firstly, it identified which nanoparticle material is best for treatment; based on the DER values, gold nanoparticles were the best. Secondly, the increase in nanoparticle concentration contributed to higher DER because there are more nanoparticles uptaken by the tumor. Therefore, it is important to determine methods to assure that the majority of the nanoparticles are placed where they need to be for greater efficiency. Thirdly, it was found that as the prostate size became smaller, the DER got greater, which means that the cancer treatment is more effective for treating smaller prostates/tumors. Fourthly, as the pelvis decreased in size, the greater the DER value. This reflects that individuals with slimmer pelvic sizes would have better results when undergoing nanoparticle-enhanced radiotherapy. Fifthly, lower energy photon beams resulted in greater DER, which means that when performing the treatment, going with a 6 MV beam will be more effective in treating the prostate. Lastly, the Monte Carlo results showed that the FFF beams always output a greater DER compared to the FF beams. Understanding these trends can help improve nanoparticle-enhanced radiotherapy and provide better results in patient treatments.
